# Low-dielectric benzocyclobutenyl polysiloxane resin: spatial structure design and photosensitive patterning performance†

**DOI:** 10.1039/d4ra08985e

**Published:** 2025-03-14

**Authors:** Juan Peng, Qiuxia Peng, Li Fan, Xian Li, Jiajun Ma, Junxiao Yang

**Affiliations:** a School of Materials and Chemistry, State Key Laboratory of Environmentally-friendly Energy Materials, Southwest University of Science and Technology Mianyang 621010 China yangjunxiao@swust.edu.cn jiajunma@yeah.net; b School of Materials Science and Engineering, Sichuan University of Science & Engineering Zigong 643000 China

## Abstract

With the development of miniaturization and the high integration of semiconductor devices, higher performance requirements are put forward for polymer photoresists. In this paper, a benzocyclobutene-based polysiloxane photosensitive resin was prepared by sol–gel method, which was well patterned and cured quickly after UV light curing, with a minimum line width and line spacing of 10 μm. The benign combination of benzocyclobutene group (BCB) and polysiloxane in the resin makes the resin exhibit good low dielectric properties (*k* = 2.79) and excellent thermal properties and mechanical properties, which is different from traditional thermosetting resins.

## Abstract 2

A polysiloxane resin negative photoresist features a low dielectric constant (*k* = 2.86), excellent film-forming properties, and effective patterning capabilities.

## Graphical abstract

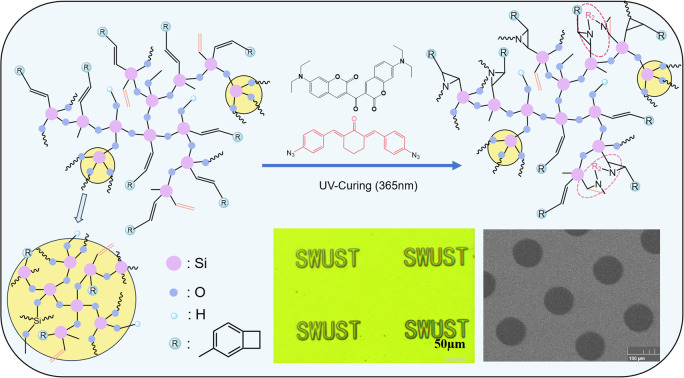

The rapid advancement of integrated circuits has led to a reduction in the feature size of semiconductor chips. The photoresist, a critical material influencing the feature size of chips, is subject to increasingly rigorous performance demands, including sensitivity, resolution, and line edge roughness.^1,2^ Generally, photoresists consist of film-forming polymers, photosensitizing agents, and solvents. Their efficacy is contingent upon attributes such as dissolution kinetics, film-forming capabilities, etch resistance, and thermodynamic properties.^3–5^

As electronic products advance towards miniaturization and heightened integration, the feature size of chips continues to diminish, leading to a substantial increase in the density of wiring. The interconnection among devices generates undesirable capacitance effects, which subsequently results in increased latency, power consumption, and crosstalk issues. The adoption of low dielectric constant (low-*K*) materials in place of conventional silica as interlayer dielectric materials represents an effective strategy to address these challenges.

Benzocyclobutene (BCB), as a whole hydrocarbon compound, is only heated to make its quaternary ring and undergo a ring-opening cross-linking reaction. After heat curing, a thermosetting resin with low dielectric properties is obtained. After curing, the simple and symmetrical structure results in low polarity within the polymer molecule after crosslinking and curing, and the resin has a low dielectric constant, which can be widely used in large-scale integrated circuits, electronic packaging, and lithography.^6–8^

Photoresists have limitations regarding resolution, sensitivity, and line edge roughness.^9–11^ To solve the problem, researchers have introduced inorganic components into photoresist components to form organic–inorganic hybrid photoresists, which combine the characteristics of organic and inorganic materials and have good development prospects.^12–14^ Organic–inorganic hybrid materials have attracted much attention due to their combination of the advantages of organic and inorganic materials.^15–18^ They can provide high thermal and chemical stability, and mechanical properties similar to those of inorganic materials. Polysiloxane is a typical representative of organic–inorganic hybrid materials because it combines organic material processability and the excellent performance of inorganic materials. The organic and inorganic Si–O–Si components in polysiloxanes constitute their excellent dielectric properties, strong heat resistance, and fairly low surface-free energy.^19–24^ Hikaru Sugita *et al.*^25^ prepared a styrene-based siloxane photoresist that formed a thin micropattern after i-line exposure and alkaline development, resulting in a transparent film with significant heat resistance. Free radicals produced by photoinitiators induce polystyrene functional groups to polymerize in photoresist films to form micropatterns. Seunghyun Jang *et al.*^26^ synthesized a polysiloxane resin-based polysiloxane binder using a sol–gel reaction and studied the photoresist properties of the silicone resin, which exhibited thermal stability at high temperature (400 °C), transmittance at 400 nm, and outgassing at 400 nm for the application of PDL in OLED devices.

In this paper, a benzocyclobutene-based polysiloxane photosensitive resin was prepared by sol–gel method (Fig. 1(A)), and its photosensitivity, thermal properties, and low dielectric properties were studied. Polysiloxane resin has a macromolecular dendritic branched structure, which combines the inorganic Si–O–Si structure and the organic group BCB in the resin benignly, so the resin has good comprehensive properties. The resin synthesis method is simple, the reaction conditions are mild, the pattern is smooth after light curing, and the edge is clear. The photothermal curing resin prepared in this paper has good thermal stability (*T*_5_ (the temperature of 5% weight loss) > 507 °C, in nitrogen) and low dielectric properties (*k* = 2.79).

**Fig. 1:**
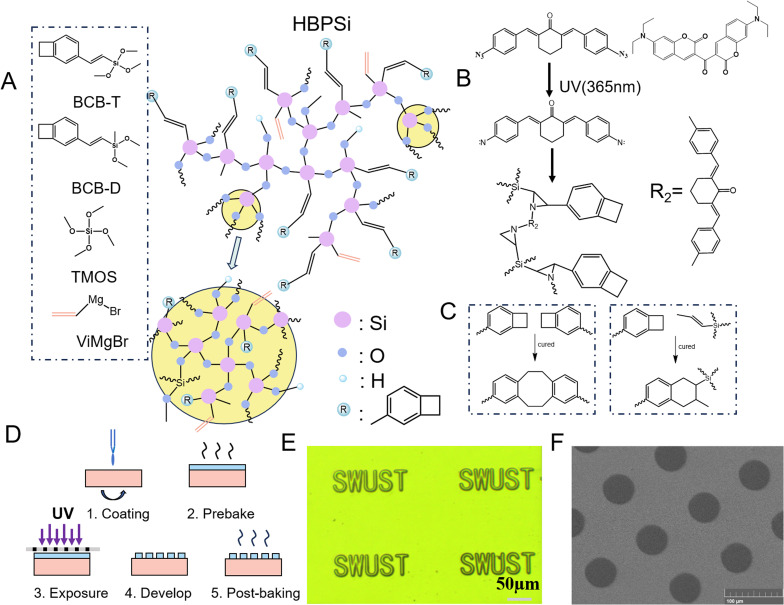
(A) Synthetic route of hybrid polysiloxane resin; (B) photocuring mechanism of photosensitive resin; (C) BCB curing flow chart; (D) photocuring flow chart; (E) lithographic pattern under an optical microscope; (F) lithographic pattern under SEM.

In the course of the study, the properties of polysiloxane resins (HBPSi-1/HBPSi-2/HBPSi-3) prepared by BCB-T and BCB-D at different molar ratios (1 : 4/1 : 9/1 : 19) were studied,^27^ and the specific performance data are shown in Fig. 3(F). The results show that the prepared polysiloxane resin HBPSi-1 has a high degree of cross-linking, good patterning effect, and good low dielectric, mechanical, and thermal properties. On this basis, the proportion of raw materials of polysiloxane resin HBPSi-1 with good comprehensive performance was selected and co-hydrolyzed with TMOS to prepare polysiloxane resin HBPSi.

Benzocyclobutene-based organic–inorganic polysiloxane resins were prepared by the sol–gel method. Their structures were characterized by nuclear magnetic hydrogen spectroscopy (^1^H NMR) and carbon spectroscopy (^13^C NMR), and detailed steps were provided in the ESI (Fig. S1 and S2).† At the same time, transmission electron microscopy demonstrated the polymer resin's microscopic morphology (Fig. S3†). A large number of small polymer particles with a particle size of about 50 nm can be seen, as well as a small number of polymer particles formed by aggregation of small particles.^28,29^ These polymer particles may be inorganic SiO_2_ or organic–inorganic hybrid polysiloxane.

After 60 seconds of light-curing of the photosensitive resin under a UV lamp with a wavelength of 365 nm, the resulting lithography patterning is shown in the figure. Fig. 1(E) is a picture taken under an optical microscope, in which the English letters SWUST with a line width and line spacing of 10 μm can be seen, the edges of the pattern are neat and clear, and there is no residual glue residue, and there is no adhesion between the patterns; Fig. 1(F) is an image taken under a SEM with a complete pattern, clear edges, and no adhesion between the patterns. The results show that the polysiloxane photosensitive resin has good lithographic patterning properties. The presence of TMOS increases the degree of cross-linking of the resin, which makes the resin cure quickly. The TMOS self-condensation agglomerated particles in the resin have no patterning properties.

A possibility analysis of the UV light crosslinking mechanism (Fig. 1(B)) showed that 3,3-carbonyl bis (7-diethylamino coumarin) absorbed photons under UV light, and the photons reacted with 2,6-bis(4-azophenylmethylene) cyclohexanone to transition from the ground state to the excited state of the single triplet state, forming carbonyl and amine radicals, which reacted with the double bonds in the polymer resin to form a nitrogen ternary ring, which made the resin cross-link and solidify to form a complex pattern insoluble in the developer.

Fourier transform infrared spectroscopy characterized the functional group changes of organic–inorganic hybrid photosensitive resins during light curing, as shown in Fig. 2(A). As the light-curing time increased, the azide absorption peak gradually decreased. The double-bond absorption peak at 985 cm^−1^ also weakened, but this was obscured by the Si–O–Si absorption band, making the peak shape less distinct. Fig. 2(B) shows the absorption peaks of different cured resins under FTIR spectroscopy, and the absorption peak at 2926 cm^−1^ is the C–H expansion vibration of the methylene group of the quaternion ring in the BCB group.

**Fig. 2:**
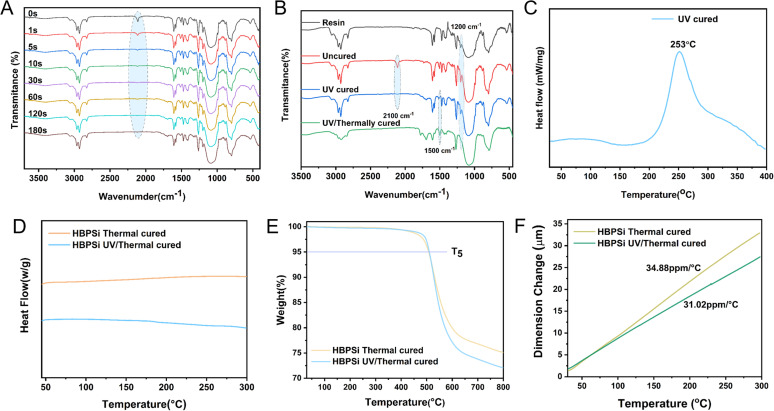
(A) Fourier infrared spectra of photosensitive resin under different illumination times; (B) FT-IR infrared spectra of resin in different curing states; (C) DSC curve of resin; (D) DSC curves of thermos-curing and photothermal-curing resins; (E) TGA curves of thermo-curing and photothermal-curing resins; (F) TMA curves of thermo-curing and photothermal-curing resins.

The absorption peaks of 1607 cm^−1^ and 1579 cm^−1^ are the backbone vibrations of the benzene ring in the BCB group. The absorption peak at 1472 cm^−1^ is the in-plane expansion vibration of C–H on the BCB quaternary ring, and the absorption peak at 1200 cm^−1^ is its out-of-plane bending vibration. The characteristic absorption peaks between 1200 and 1000 cm^−1^ are the inorganic Si–O–Si structures. The absorption peak of 989 cm^−1^ is the C–H out-of-plane bending vibration of the vinyl group in the resin, indicating that the vinyl double bond between the silicon atom and the BCB group is a trans structure. After the addition of a photoinitiator, the azide absorption peak at 2100 cm^−1^ appeared, the absorption peak disappeared after light curing, and the absorption peak of the C=C bond weakened, indicating the occurrence of a photocrosslinking chemical reaction. After photothermal curing, a new absorption peak appeared at 1500 cm^−1^, which was related to the ring strain released by the quaternary ring in the BCB during curing. A new absorption band appeared at 2839 cm^−1^, which was related to the C–H vibration of six-membered cyclic radicalization formed by BCB loop opening. The characteristic absorption peak of vinyl at 990 cm^−1^ disappeared after heat curing. The change of the absorption peak of the double bond indicates the smooth progress of light curing, and the change of the characteristic absorption peak of the BCB quaternary ring suggests that the cross-linking reaction occurs in the opening of the quaternary ring, and the polysiloxane resin completes the heat curing.

The curing behavior of HBPSi resin was characterized by differential scanning calorimetry (DSC). Fig. 2(C) is the DSC curve of a UV-cured HBPSi resin at a heating rate of 20 °C min^−1^ in an N_2_ atmosphere. As shown in the figure, the exothermic peak of the photosensitive resin indicates the temperature of thermal curing, with an initial temperature of 199 °C and a maximum exothermic peak of 253 °C, which suggests that the BCB group undergoes a ring-opening crosslinking reaction during the thermal curing process, which further improves the degree of crosslinking of the polysiloxane resin. Fig. 2(D) is the DSC curve of the heat-curing resin and the photothermal-curing resin, the heat-curing resin, and the photothermal-curing resin have no exothermic peak within 300 °C. The curves are flat, indicating that under the set curing conditions, all the BCB quaternary rings in the resin are open to forming six-membered or eight-membered rings, and the resin is completely cured (Fig. 1(C)).

Thermal stability is also an important indicator for testing the performance of low-dielectric materials. Fig. 2(E) shows the TG curve of the cured resin in the N_2_ atmosphere (with a heating rate of 20 °C min^−1^) and exhibits good thermal stability, which is significantly higher than that of DVSBCB (440 °C) resin. According to the *T*_5_ value, it can be seen that the *T*_5_ of the heat-curing resin is 507 °C, the *T*_5_ of the photothermal-curing resin is 510 °C, and the cured resin shows good thermal stability. This is attributed to the polymer with a high cross-linking network structure formed by the unique thermal ring-opening polymerization mechanism of the BCB group in the resin structure, which gives the polymer excellent thermal stability.

The coefficient of thermal expansion results are shown in Fig. 2(F). The results showed that the coefficient of linear thermal expansion of HBPSi heat-curing resin was 34.88 ppm °C^−1^ from room temperature to 300 °C (heating rate of 10 °C min^−1^), and that of photothermal-curing resin was 31.02 ppm °C^−1^. The results show that the polysiloxane resin has good thermal expansion performance, which is attributed to the cross-linking reaction of the ring opening of the BCB group during the curing process, which cross-links the molecular segments that are far away and improves the cross-linking degree of the resin, and the resin is not easy to deform at high temperature after curing.

A nanoindentation instrument tested the mechanical properties of the homogeneous and inorganic hybrid resin after curing, and the hardness and modulus of the organic–inorganic hybrid resin HBPSi were evaluated by measuring multiple indentation points in continuous stiffness mode. The test results are shown in Fig. 3(A–C) with a hardness of 0.75 GPa for the heat-curing resin, 9.50 GPa for Young's modulus, 0.94 GPa for the photothermal-curing resin, and 12.20 GPa for Young's modulus. The two cured resins have a high modulus of elasticity and hardness. The presence of a significant number of BCB groups with a rigid structure in the resin contributes to its high hardness and enhances its mechanical properties following heat curing.

**Fig. 3:**
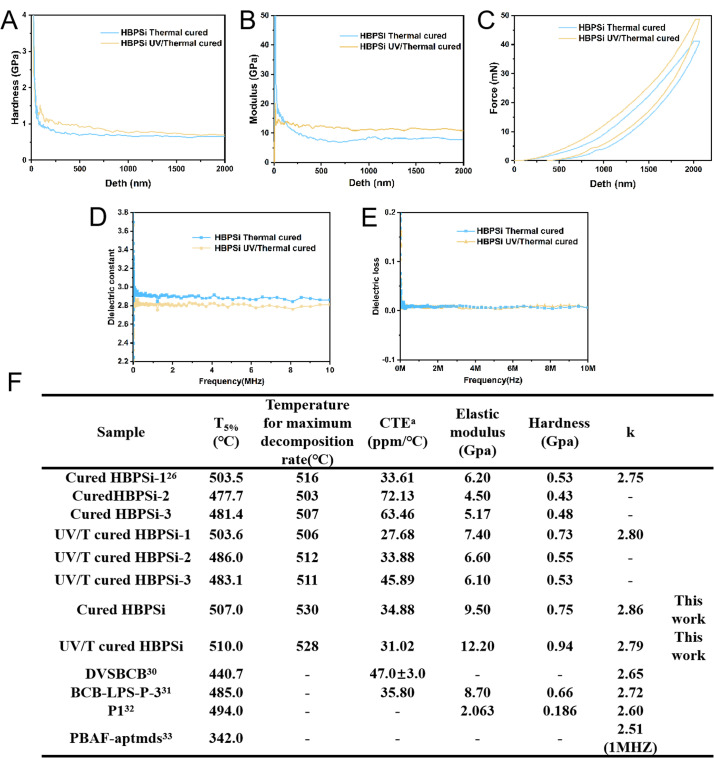
(A–C) Modulus, hardness, and load–unload curves of the thermoformable resins; (D) dielectric constant of cured resin change with frequency; (E) dielectric loss of cured resin change with frequency; (F) comparison of the properties of resins.^27,30–33^

After thermal and photothermal curing, the resin was ground into a regular cylinder, and its dielectric constant performance was tested at room temperature using a precision impedance meter in the range of 10 MHz. The dielectric constant as a function of frequency is shown in Fig. 3(D and E).

The dielectric constants of the two curing values in this range are relatively stable, and the dielectric constants of the thermal-curing and photothermal-curing resins are calculated to be 2.86 and 2.79, respectively. The presence of more BCB groups in the resin facilitates the ring-opening process after heat curing, significantly enhancing the cured resin's cross-linking degree and porosity. This contributes to the excellent dielectric properties exhibited by the cured material. Additionally, the substantial quantity of low-grade Si–O and Si–C bonds present in the polysiloxane portion of the resin further enhances its electrical properties. The harmonious combination of these two components results in a thermosetting resin that boasts a commendable low dielectric constant. Because the organic–inorganic hybrid resin contains many highly polar organic–inorganic Si–O–Si structures, the dielectric constant is relatively high.

Fig. 3(F) shows that HBPSi resin has good thermal stability, mechanical properties, and low dielectric properties compared with similar organic–inorganic hybrid materials. Compared with HBPSi (1–3) in the previous work, HBPSi resin has improved thermal stability and mechanical properties, and a large number of siloxane groups have little effect on the low dielectric properties.

In summary, a benzocyclobutene-based organic–inorganic hybrid polysiloxane resin was prepared by sol-coagulation method, and the Grignard reagent of vinyl magnesium bromide was used to reduce the negative effects of residual Si–OCH_3_ in the resin and introduce more vinyl double bond groups, which made the light curing process faster and simpler. The branched structure of the resin gives it good thermal stability and dielectric properties, and the addition of TMOS can improve the degree of resin cross-linking and accelerate the light-curing rate while reducing the economic cost. Optical microscopy and scanning electron microscopy characterized the good lithography patterning properties of the photosensitive resin, nuclear magnetic hydrogen spectroscopy, carbon spectroscopy, and Fourier transform infrared spectroscopy proved the successful preparation of the resin, the light curing kinetics was monitored by Fourier transform infrared spectroscopy, and the results of transmission electron microscopy showed the microstructure of the resin. This photosensitive resin has good patterning properties, low dielectric constant (*k* = 2.79), high thermal stability (*T*_5_ = 510 °C), and excellent mechanical properties, and has a good development prospect in the field of UV curing.

## Data availability

The data supporting this article have been included in the ESI.†

## Author contributions

Juan Peng, Jiajun Ma, and Junxiao Yang designed and engineered the samples; Juan Peng performed the experiments; Juan Peng, Li Fan, and Xian Li assisted with the sample testing; Juan Peng, Qiuxia Peng, Jiajun Ma, and Junxiao Yang helped with manuscript writing. All authors contributed to the general discussion.

## Conflicts of interest

There are no conflicts to declare.

## Supplementary Material

RA-015-D4RA08985E-s001
