# Supplementary material for: Low-dielectric benzocyclobutenyl polysiloxane resin: spatial structure design and photosensitive patterning performance
Source: RSC Adv. 2025 Mar 14;15(10):7956–61. doi: 10.1039/d4ra08985e (PMC11907431; doi:10.1039/d4ra08985e)
Supplement: RA-015-D4RA08985E-s001 [file RA-015-D4RA08985E-s001.pdf]

Supplementary Information (SI) for RSC Advances.
This journal is © The Royal Society of Chemistry 2025

Low-dielectric benzocyclobutenyl polysiloxane resin: spatial structure design and photosensitive patterning performance

Juan Peng[a]; Qiuxia Peng[b]; Li Fan[a]; Xian Li[a]; Jiajun Ma[a]*; Junxiao Yang[a]*

a. School of Materials and Chemistry and State Key Laboratory of Environmentally-friendly Energy Materials, Southwest University of Science and Technology, Mianyang 621010, China.

b. School of Materials Science and Engineering, Sichuan University of Science & Engineering, Zigong 643000, China.

† Corresponding Authors: Junxiao Yang (yangjunxiao@swust.edu.cn), Jiajun Ma (jiajunma@yeah.net)

CONTENTS

Page2-Page3: Experimental: materials, measurements and synthesis

Page4: Table S1: Material ratio of **HBPSi** resin

Page5: Fig. S1: ^{1}H NMR and ^{13}C NMR spectrum of **HBPSi**

Page6: Fig. S2: Transmission electron microscope image of **HBPSi** resin

Page7: Fig. S3: DTG spectrum of thermal curing and photothermal curing resin

■ Experimental

● Materials

Tetramethoxysilane (TMOS, >99%) and vinyl magnesium bromide (1.0 M solution in 2-MeTHF) are from Shanghai Titan Technology Co., Ltd.; concentrated hydrochloric acid, toluene, and anhydrous sodium sulfate, all from Chengdu Kelong Chemical Co., Ltd.; Tetrahydrofuran (THF, >99.8%), Beijing J&K Technology Co., Ltd.; Ultrapure water (18.25MΩ *cm(25°C)),lab-made;4-(trimethoxyvinyldimethylsilyl)benzocyclobutene(BCB-T),4-(dimethoxyvinyldimethicyl)benzocyclobutene (BCB-D)[1], lab-made.

● Measurements

Nuclear magnetic resonance Spectrometer (Bruker Avance-600), Bruker, Switzerland; Infrared Absorption Spectrometer (NICOLET-5700), Nicolet Instruments, USA; Differential scanning calorimeter (TA Q200), TA Corporation, USA; Agilent U9820A Nano Indenter G200, Agilent Technology Co. Ltd. Static Thermo-mechanical Analysis (USA-TA-Q400), TA Corporation, USA; Scanning electron Microscope (ZEISS EVO 18), Carl Zeiss, Germany; Optical Microscope (ISH500), Shanghai Rectangular Optics; Glue leveling machine (EZ4), Jiangsu Leibo Instrument; Precision Impedance Analyzer (HR 4294 LCR), Agilent Technologies Ltd.

● Synthesis

1. Preparation of benzo cyclo butenyl polysiloxane resins

1 g (4 mmol) BCB-T, 3.74 g (16 mmol) BCB-D, 0.60 g (4 mmol)) TMOS and 0.246 g of ultrapure water was added to a 100 mL eggplant-shaped reaction flask, 30.65 μL of concentrated hydrochloric acid was added dropwise, stirred for 1 h at room temperature, raised to 50 ^{o}C and continued to stir for 5 h, and then 0.246 g of ultrapure water and 30.65 μL of concentrated hydrochloric acid were stirred dropwise for 5 h. The water pump removes small molecules such as hydrochloric acid, water, and by-products in the reaction system to obtain a light-yellow viscous polymer resin. 3 mL of tetrahydrofuran dissolving resin was added, and 15 mL (15 mmol) of vinyl magnesium bromide solution was added drop by drop at room temperature and in an N_2 atmosphere, and the reaction system was exothermic during the reaction, a large number of bubbles were generated, and the gas was released. After 1 h, the ethanol was quenched, and the THF solution in the spin-dried system was brownish-yellow viscous. Toluene was added to dissolve the polymer,

the organic system was washed with water, repeated three times, the upper organic solution was obtained by separating, the anhydrous sodium sulfate was dried as the organic solution, and the light-yellow viscous resin HBPSi was obtained by spinning.

2. Preparation of polysiloxane photosensitive resin

The benzocyclobutene polysiloxane resin (1 g), add it to a 3mL brown vial, weigh and add 3wt% photoinitiator 2,6-bis-(4-azophenylmethylene) cyclohexanone (BAC), 1wt% 3,3'-carbonylbis(7-diethylaminocoumarin), and then add the solvent toluene: cyclopentanone (3:1) to the brown vial, and dissolve it ultrasonically to obtain a photosensitive solution; The photosensitive solution was dropped on the glass sheet for spin coating, the rotation speed was 3000 rpm, and the rotation was 60 s to obtain the photosensitive film (Uncured film), which was put into an oven at 80 °C and baked for 10 minutes; Evaporate the solvent.

The photosensitive solution was dropped on a glass sheet for spin coating at 3000 rpm and rotated for 60 seconds to obtain an uncured film, which was placed in an oven at 80 °C for 10 minutes. After the solvent evaporated, the 365 nm UV-LED point light source was used to expose the photosensitive film through the photomask, and the exposed area was cross-linked and solidified after 60 s of exposure, and it was difficult to dissolve in the developer solution. In contrast, the unexposed area was soluble in the developer. After the development of the developer (cyclohexanone: petroleum ether = 4:1), the pattern (UV-cured film) consistent with the photomask was obtained.

Table S1 Material ratio of **HBPSi** resin

Sample	BCB-T: BCB-D	TMOS	Si-OCH_3:H_2O	ViMgBr:Si-OCH_3 (D+T)	HCl: (D+T)	Solvent
HBPSi	1: 4	1	1: 2	1: 4	3.0 %	No solvents

Note: All ratios are molar ratios

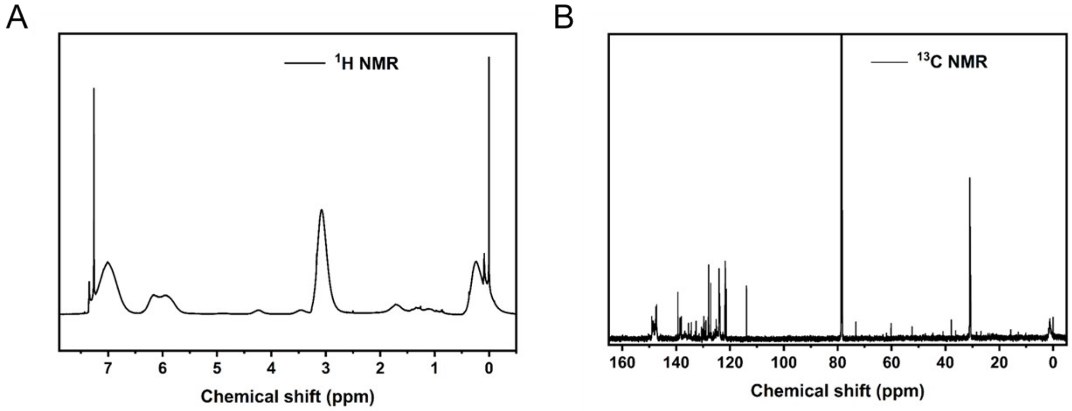

Fig. S1 (A) and (B) ^{1}H NMR and ^{13}C NMR spectrum of **HBPSi** resin

In ^{1}H NMR (Fig.S1 (A)) 0-0.5 ppm is the chemical shift of Si-CH_3, around 3 ppm is the chemical shift of the benzocyclobutylene quaternary ring, 5.5-6.5 ppm is the chemical shift of the vinyl group in the resin, the content of the end vinyl group produced during the end-capping process is less, so its chemical shift may be covered in this range, and 6.5-7.3 ppm is the chemical shift of the benzene ring in the resin. Fig.S1 (B) is a ^{13}C NMR spectrum of HBPSi resin, and the chemical shift of C of Si-CH3 in the polymer resin is about 1.24 ppm. The chemical shifts of C of the benzocyclobutene quaternary ring are approximately 30.75 ppm and 30.97 ppm; The chemical shifts of vinyl C in polymers are approximately 120.18 ppm and 122.53 ppm; 113.89 ppm and 121.79 ppm were the chemical shifts of C of vinyl magnesium bromide-capped vinyl, respectively. The chemical shifts of C in the benzene ring were 124.05 ppm, 138.06 ppm, 139.34 ppm, 147.23 ppm, 147.56 ppm, and 149.02 ppm, respectively. The results showed that the branched organic-inorganic hybrid polysiloxane resin was successfully prepared.

The Grignard reagent of vinyl magnesium bromide reacts with the residual Si-OCH_3 in the resin to form a silica vinyl group. During the aqueous washing process of siloxane, part of the silicon is converted to Si-OH, and Grignard reagent vinyl magnesium bromide will also react with it [2]. Still, some Si-OH will remain in the polymer resin, and the residue is small, so it is not obvious in ^{1}H NMR.

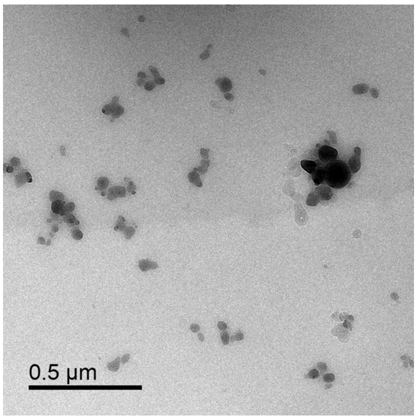

Fig. S2 Transmission electron microscope image of **HBPSi** resin

The microstructure of polysiloxane resins was characterized by transmission electron microscopy as shown in Fig. S2. A large number of small polymer particles with a particle size of about 50 nm can be seen, as well as a small number of polymer particles formed by aggregation of small particles[3,4]. These polymer particles may be inorganic SiO_2 or organic-inorganic hybrid polysiloxanes.

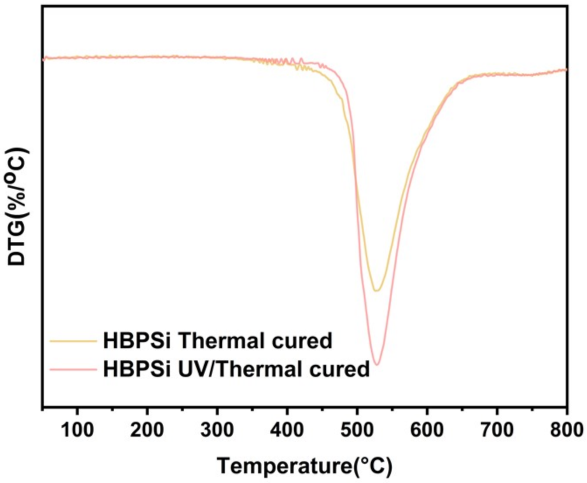

Fig. S3 DTG spectrum of thermal curing **HBPSi** resin

Reference

1 Juan Peng, Chao Guo, Xinyu Hu, et. al. *RSC Appl. Polym.*, 2024, 2, 606-611.

2 Lijuan He, Zongbo Zhang, Xiaogan Yang, et al. *Polymer International,* 2015, 64(8), 979-985.

3 Qiu xia Peng, Huan Hu, Yue ting Deng, et al. *Journal of Materials Chemistry C*, 2022, 10: 9106.

4 Qiuxia Peng, Jiajun Ma, Ji Wu, et al. *Polym Chem*, 2023, 14, 3446-3452.
